# Functional divergence and symbiotic significance of nitrate reductase isoforms in *Medicago truncatula*

**DOI:** 10.1093/plphys/kiag377

**Published:** 2026-06-16

**Authors:** Marc Bosseno, Alexandre Demba, Natasha Horta Araújo, Dominique Colinet, Abir Israel, Marie Pacoud, Mickael Maucourt, Yassine El Fazaa, Daniel Jacob, Marc Lepetit, Dominique Rolin, Renaud Brouquisse, Alexandre Boscari

**Affiliations:** Institut Sophia Agrobiotech, UMR INRAE 1355, Université Côte d’Azur, CNRS, Sophia Antipolis France; Institut Sophia Agrobiotech, UMR INRAE 1355, Université Côte d’Azur, CNRS, Sophia Antipolis France; IRD, Plant Health Institute of Montpellier (PHIM), UMR IRD/SupAgro/INRAE/UM/CIRAD, Montpellier, France; Institut Sophia Agrobiotech, UMR INRAE 1355, Université Côte d’Azur, CNRS, Sophia Antipolis France; Institut Sophia Agrobiotech, UMR INRAE 1355, Université Côte d’Azur, CNRS, Sophia Antipolis France; Institut Sophia Agrobiotech, UMR INRAE 1355, Université Côte d’Azur, CNRS, Sophia Antipolis France; Biologie du Fruit et Pathologie, Université de Bordeaux, INRAE, Villenave d’Ornon France; Bordeaux Metabolome, MetaboHUB, PHENOME-EMPHASIS, Villenave d’Ornon France; Institut Sophia Agrobiotech, UMR INRAE 1355, Université Côte d’Azur, CNRS, Sophia Antipolis France; UR1268 BIA, BIBS Facility INRAE, Nantes, France; Institut Sophia Agrobiotech, UMR INRAE 1355, Université Côte d’Azur, CNRS, Sophia Antipolis France; Biologie du Fruit et Pathologie, Université de Bordeaux, INRAE, Villenave d’Ornon France; Bordeaux Metabolome, MetaboHUB, PHENOME-EMPHASIS, Villenave d’Ornon France; Institut Sophia Agrobiotech, UMR INRAE 1355, Université Côte d’Azur, CNRS, Sophia Antipolis France; Institut Sophia Agrobiotech, UMR INRAE 1355, Université Côte d’Azur, CNRS, Sophia Antipolis France

## Abstract

Nitrate reductase (NR) is a key enzyme in nitrate assimilation, yet its function within nodules remains poorly understood. In *Medicago truncatula*, 3 NR genes, *MtNR1*, *MtNR2*, and *MtNR3*, exhibit distinct evolutionary origins and regulatory features. Phylogenetic analyses indicate that *NR3*-type genes, originated from a duplication of *NR1* within the inverted repeat-lacking clade (IRLC) legumes, have lost the conserved phosphorylation sites critical for post-translational regulation. To assess the functional significance of these isoforms, we characterized single and double *nr* mutants obtained through *Tnt1* transposon insertion under nitrate nutrition and during symbiosis. MtNR1 is the primary contributor to total NR activity, with *nr1* and *nr2* mutants retaining around 10% and 30% of wild-type levels, respectively. The *nr1/nr2* double mutant shows an almost complete loss of NR activity and fails to survive under nitrate supply, demonstrating the essential and non-redundant roles of both isoforms. Under symbiotic conditions, single mutants displayed normal nodulation, whereas nodule development was nearly abolished in the double mutant despite continued *MtNR3* expression. In addition to its role in nitrogen assimilation, single *nr* mutants showed increased sensitivity to hypoxic stress and impaired recovery of nitrogen fixation, revealing a role for NR in nodule energy metabolism through the phytoglobin–NO respiration pathway. We propose that the combined loss of NR1 and NR2 disrupts NO cycling linked to mitochondrial electron transport, thereby compromising the energy balance required for symbiosis under microoxic conditions. This work provides a framework to investigate NR diversification in legumes and opens perspectives for improving nitrogen fixation under environmental constraints.

## Introduction

In land plants, yeasts, algae, and fungi, nitrate reductase (NR) is a key enzyme for nitrogen acquisition. The enzyme catalyses the reduction of nitrate (NO_3_^−^) to nitrite (NO_2_^−^). Nitrite is then reduced to ammonia (NH_4_^+^) by nitrite reductase (NiR) before being assimilated into the amino acids and nitrogen compounds of the cell (Campbell 1988; Meyer and Stitt 2001). Beyond this primary role, NR also exhibits nitrite:NO reductase (Ni-NR) activity, enabling the further reduction of nitrite to nitric oxide (NO), under hypoxic conditions, high nitrite concentrations, or acidic environments (Rockel et al. 2002). NR is a homodimer consisting of 2 approximately 100 kDa polypeptide chains. It is responsible for the first intracellular and rate-limiting step in nitrate assimilation. Each chain binds 3 cofactors in individually folded domains, and the enzyme operates as an internal electron transport chain (Campbell 1988). The C-terminal domain contains a flavin adenine dinucleotide (FAD) cofactor and accepts 2 electrons from NADH or NADPH. It then transfers these electrons to the middle domain, which carries a b5-type cytochrome heme. The electrons are then transported to the catalytic site, which contains a molybdenum cofactor (Moco) in the N-terminal domain, where substrate reduction takes place. In addition to the regulation of steady-state levels of NR protein at the transcriptional and protein turnover levels (Hoff et al. 1994), post-translational modification of NR by protein phosphorylation is an important regulatory mechanism (Douglas et al. 1995; Bachmann et al. 1996; Su et al. 1996; Lillo et al. 1997, 2003).

Beyond their canonical role in nitrogen assimilation, NRs also contribute to an alternative metabolic route that sustains energy production under low-oxygen conditions: the phytoglobin–NO respiration (PNR) pathway. In this process, NR reduces nitrate to nitrite, which is subsequently transported to mitochondria and reduced to nitric oxide (NO) by components of the mitochondrial electron transport chain. NO then diffuses back into the cytosol, where it is oxidized to nitrate by phytoglobins, thereby completing a cyclic process that supports ATP generation and maintains cellular redox balance during hypoxia (Igamberdiev 2004; Igamberdiev and Hill 2009). This mechanism operates across a range of plant tissues, underscoring a broader role for NRs in sustaining energy homeostasis in oxygen-limited environments.

In the Rosid clade, leguminous plants have developed a mutually beneficial relationship with soil bacteria (rhizobia) that enables them to convert atmospheric nitrogen (N_2_) to ammonia (NH_4_^+^) through the activity of nitrogenase (Postgate 1982). In exchange for fixed nitrogen, plants provide an ecological niche to the bacteria for their development and carbon nutrients for their functioning in newly formed organs called nodules (Udvardi and Day 1997; Terpolilli et al. 2012). In legume nodules, the bacterial nitrogenase produces NH_4_^+^ instead of the NR-NiR pathway and supplies it to the plant. However, numerous studies have shown high levels of NR expression and activity in symbiotic nodules (Streeter 1985a, 1985b; Arrese-Igor et al. 1990; Silveira et al. 2001), and the localization of NR mRNA within the infected regions of pea root (Kato et al. 2003) and *M. truncatula* nodules (Horchani et al. 2011) raises the question of what NR can do in the N_2_-fixing symbiosis, since reduced nitrogen is provided by nitrogenase. Despite the identification of *NR* mutants in legumes (Feenstra and Jacobsen 1980; Ryan et al. 1983), no study fully elucidated the significance of these enzymes in the symbiotic nitrogen fixation process. The presence of 3 *NR* genes in the *M. truncatula* genome (Puppo et al. 2013) raises the question of their respective roles in nitrate-fed and symbiotic plants. During the *M. truncatula–Sinorhizobium meliloti* interaction, *MtNR1* and *MtNR2* expression peaks coincide with NO production, which is abolished by tungstate or *NR1/NR2* RNAi knockdown (Berger et al. 2020b). Recent studies using RNAi-mediated knockdown or pharmacological inhibition of NR by tungstate indicate that NR activity may be essential for energy metabolism and N_2_ fixation in mature nodules (Horchani et al. 2011; Aridhi et al. 2020; Chammakhi et al. 2022) and could contribute to ATP regeneration through its involvement in the phytoglobin–NO respiration pathway under hypoxic conditions (Chammakhi et al. 2025). Despite the direct provision of ammonium by nitrogenase in legume nodules, NR activity is maintained at significant levels, yet the specific contributions of individual NR isoforms to NO production, energy metabolism, and hypoxia resilience remain poorly understood. This study aims to uncover how MtNR1, MtNR2, and MtNR3 integrate nitrogen metabolism, NO signaling, and bioenergetic homeostasis in nodules.

In the present work, we first conducted a phylogenetic analysis to explore the evolutionary relationships among NR protein sequences across various orders within the Rosids clade. To dissect the respective role of 3 NR isoforms in *M. truncatula*, we analyzed *Tnt1* retrotransposon-tagged *nr* mutants and generated a double *nr1/nr2* mutant. These lines were then phenotypically characterized under nitrate-supplemented conditions, during the nodulation process, and in response to hypoxic stress.

## Materials and methods

### Plants growth and inoculation conditions

Seeds of wild-type *M. truncatula* ecotype R108 were scarified, surface-sterilized, and germinated as described in Del Giudice et al. (2011). Germinated seedlings were transferred to square plates containing Fahräeus medium with 0.2 mM NH_4_NO_3_, individual pots, or hydroponic systems. Plants in individual pots (Pots PP polypropylene, 9*9*9 cm, Soparco), containing a mixture of vermiculite and perlite (2:1, v/v), were watered every 2 to 3 d, 2 times with water for 1 time with 1 g.l^−1^ of nutrient solution. In hydroponic culture, 6 d-old seedlings were transferred to aerated basal nutrient solution HY (Lambert et al. 2020) supplemented with 1 mM NH_4_Cl to support growth in nitrate reductase-deficient lines. After 1 wk, the solution was renewed and either supplemented with 1 mM KNO_3_ or inoculated with *Sinorhizobium meliloti* (10^7^ cfu ml^−1^) and 10 µM of KNO_3_. All plants were maintained at 20 to 23 °C under a 16 h light/8 h dark photoperiod. Plate and pot-grown plants were inoculated 5 d after transfer with *S. meliloti* 2011 strain at OD600 0.5 as described in Del Giudice et al. (2011).

Homozygous *M. truncatula Tnt-1* insertion mutants in the R108 genetic background, NF15469 (insertion in *MtNR1)*, NF14768 (insertion in *MtNR2*) and NF11190 (insertion in *MtNR3*) were obtained from the Noble Research Institute (Ardmore, OK, United States) (Tadege et al. 2008). *Tnt1* insertion sites were confirmed using gene-specific primers (Table S1) in combination with LTR4 and LTR5 primers (Ratet et al. 2010). The NF15469 (6 *Tnt1* insertions), NF14768 (5 *Tnt1* insertions), and NF11190 (20 *Tnt1* insertions) were backcrossed 3, 3, and 4 times, respectively, to clean up the genetic background of these mutants following the protocol described by Bosseno et al. (2020). To generate the *nr1/nr2* double mutant, *nr1* and *nr2* single mutants were crossed. Due to severe developmental defects, flowering and seed production could not be achieved in the *nr1/nr2* double mutant line, even under various N regimes (ammonia, urea, etc.). The seedlings of the *nr1/nr2* genotype used in this work were obtained by self-fertilization of the *nr1/nr1_nr2/NR2* genotype line, derived from the previous cross between *nr1* and *nr2* mutants. As expected from Mendelian segregation, 25% of the progeny carried both mutant alleles (Table 1). These *nr1/nr2* seedlings were identified by PCR genotyping and analyzed alongside 2 control siblings (*nr1_Sibl1* and *nr1_Sibl2*) for phenotypic and molecular comparisons (nomenclature detailed in Table 2, Figure S11).

**Table 1:** Punnett square genotype for the self-fertilisation of the line knockout for the *NR1* gene and heterozygous on *NR2* gene allele. Expected frequencies of each genotypic category are indicated in parentheses.

	*nr1/nr2*	*nr1/NR2*
*nr1/nr2*	*nr1/nr2 (1/4)*	*nr1_Sibl2 (nr1/nr1_nr2/NR2) (1/4)*
*nr1/NR2*	*nr1_Sibl2 (nr1/nr1_NR2/nr2) (1/4)*	*nr1_Sibl1 (nr1/nr1_NR2/NR2) (1/4)*

**Table 2:** Nomenclature of mutant lines used in this manuscript.

*Name*	*Genotype*	*^o^*
*nr1*	*nr1-1^ko^*	Knockout for the *NR1* gene
*nr2*	*nr2-1^ko^*	Knockout for the *NR2* gene
*nr3*	*nr3-1^ko^*	Knockout for the *NR3* gene
*nr1_Sibl1*	*nr1^ko^*	Knockout for the *NR1* gene with no Tnt1 insertion in the *NR2* gene
*nr1_Sibl2*	*nr1^ko^_nr2^HT^*	Knockout for the *NR1* gene and heterozygous on *NR2* gene allele
*nr1/nr2*	*nr1/nr2-1^ko^*	Knockout for the *NR1 and NR2* gene

### Phylogenetic analysis of NR protein sequences

NR protein sequences were initially obtained from BLASTP searches against the refseq_protein database at NCBI (www.ncbi.nlm.nih.gov/) using *M. truncatula* MtNR1, MtNR2, and MtNR3 sequences as queries and an e-value threshold of 1.0e^−5^. BLASTP searches were restricted to Rosids (taxid:71275), excluding *Medicago* (taxid:3877), with a maximum number of hits of 500 on the one hand, and to Poales (taxid:71275) with a maximum number of hits of 100 on the other. The resulting sequence dataset was supplemented with NR protein sequences from Rosids obtained from an exhaustive manual search of the Phytozome genomics database (https://phytozome-next.jgi.doe.gov/), and of the UniprotKB (www.uniprot.org) and NCBI non-redundant (https://blast.ncbi.nlm.nih.gov/Blast.cgi) protein databases. A CD-HIT analysis was then performed with a similarity threshold of 95% to remove redundancy (Li and Godzik 2006). Finally, orders belonging to Rosids that contained only 1 sequence were eliminated and only 1 species was retained for each genus. A total of 121 non-redundant protein sequences, ranging from 582 to 956 amino acids and belonging to 64 species were retained for phylogenetic analysis and are listed in Table S2.

The 121 NR protein sequences were aligned using MAFFT (v7) with the auto option, which automatically selects the appropriate alignment strategy based on data size (Katoh and Standley 2013). Poorly aligned regions were removed using trimal (v1.4) with the -automated1 option, which is optimized to automatically select the best trimming method for maximum likelihood (ML) phylogenetic analysis (Capella-Gutiérrez et al. 2009). The ML phylogeny of NR protein sequences was inferred using IQ-TREE (v2) (Minh et al. 2020) with automated model selection (Kalyaanamoorthy et al. 2017). Support values were based on a Shimodaira–Hasegawa approximate likelihood ratio test (SH-aLRT) (Guindon et al. 2010) combined with an ultrafast bootstrap approximation (UFboot) with 1,000 replicates each (Hoang et al. 2018). Only support values greater than or equal to 80% and 95% for SH-aLRT and UFboot, respectively, were considered. Phylogenies were visualized and annotated using iTol (Letunic and Bork 2021).

### Search for the gene encoding MtNR1 in IRLC clade species

To investigate the absence of *MtNR1* in some species of the IRLC clade, a protein-to-genome comparison using Exonerate (v2) with a score threshold of 500 (Slater and Birney 2005) was performed with the 3 *M. truncatula* NR protein sequences in the genomes of *Pisum sativum* (NCBI accession number GCF_024323335.1), *Vicia faba* (NCBI accession number GCA_948472305.1) and *Cicer arietinum* (NCBI accession number GCF_000331145.1). Meanwhile, a synteny analysis was performed between the *M. truncatula*, *P. sativum*, *V. faba* and *C. arietinum* genomes using MCScanX (Wang et al. 2012).

### Analysis of Fabales sequence phosphorylation sites

The 46 NR protein sequences from *Fabales* and *Cucurbitales* species, as well as *A. thaliana,* were aligned using MAFFT (v7) with the auto option, which automatically selects the appropriate alignment strategy based on data size (Katoh and Standley 2013). The amino acids in the hinge regions between Moco and Heme and Heme and FAD were identified using the PFAM tool (El-Gebali et al. 2019). Prediction of the phosphorylation sites within each sequence included in the alignment was conducted using MusiteDeep ((Wang et al. 2017); https://www.musite.net). Phosphorylation sites with a probability higher than 0.5 were selected and compared with predictions generated by the PlantPhos tool (Lee et al. 2011). Predicted and known *A. thaliana* phosphorylation sites were manually annotated in the alignment using Microsoft Word.

### Vectors constructions and roots transformation by *Agrobacterium rhizogenes*

The full-length cDNA of *M. truncatula MtNR2* was amplified by PCR using primers a135 and a136 (Table S1), cloned into pENTR4 vector, and transferred into the pK7WG2D vector under the control of the 35S promoter using Gateway recombination (Karimi et al. 2002). The resulting construct, pK7WG2D:MtNR2, was introduced into *Agrobacterium rhizogenes* strain Arqua1 (Quandt et al. 1993) and used to transform *M. truncatula* according to established protocols (Boisson-Dernier et al. 2001; Vieweg et al. 2004). Transgenic roots were selected 2 wks after germination based on GFP fluorescence using a Leica MZ FLIII fluorescence stereomicroscope (Leica, Wetzlar, Germany). Composite plants were transferred to Fahräeus medium supplemented with 1 mM NH_4_NO_3_, or to planters containing 5 mM NH_4_NO_3_ for complementation assays.

### Gene expression analysis

Harvested nodules and roots were frozen in liquid nitrogen and ground. Total RNA extraction was performed using the RNAzol RT reagent following the manufacturer's recommendations (Sigma-Aldrich, United States). After the DNAse treatment with RQ1 DNAse (Promega, United States), 1 µg or total RNA was used for the cDNA synthesis (GoScript Reverse Transcription System, Promega). Quantitative real-time PCRs were performed in the Agilent AriaMx System using the GoTaq qPCR Master Mix (Promega, United States). Two reference genes (MtC27 and a38) were used to normalize the transcript level. The complete list of primers is reported in Table S1. Data were quantified using the Aria Software and analyzed with RqPCRBase, an R package working on R computing environment for analysis of quantitative real-time PCR data (Rancurel et al. 2019).

### Nitrogen-fixing capacity

The nitrogen-fixing capacity of nodules was determined in vivo by measuring the reduction of acetylene to ethylene, as previously described as acetylene-reducing activity (ARA, (Hardy et al. 1968)). Nodulated roots were harvested and incubated at 30 °C for 1 h in rubber-capped tubes containing a 10% (v/v) acetylene atmosphere. Two biological replicates have been performed with 5 technical replicates. Ethylene concentrations were determined by gas chromatography (Agilent GC 6890N, Agilent Technologies, Santa Clara, CA, United States) equipped with a GS-Alumina (30 m × 0.534 mm) separating capillary column.

### Measurement of NO production

NO detection was performed as in Horchani et al. (2011) using the 4,5-diaminofluorescein probe (DAF-2, Sigma-Aldrich) or cell-trappable copper(II) fluorescent probe (CuFL2A, Strem Chemicals) with the following changes. Either nodules (20 to 30 mg fresh weight) or root segments (50 to 100 mg fresh weight) were incubated in 1 ml of detection buffer (10 mM Tris-HCl pH 7.4, 10 mM KCl) in the presence of 10 µM DAF-2 or 5 µM CuFL2A with or without 300 µM 2-(4-Carboxyphenyl)-4,4,5,5-tetramethylimidazoline-1-oxyl-3-oxide (cPTIO, Sigma-Aldrich). The production of NO was measured with a spectrofluorimeter-luminometer (Xenius, SAFAS, Monaco).

### Measurement of nitrate reductase activity

Tissue samples are ground with mortar and pestle in liquid nitrogen. The total proteins are extracted from 100 mg of powder using the following extraction buffer: 25 mM Tris HCl pH 8.5, 1 mM EDTA, 20 μM FAD, 0.04% (v/v) Triton, 10 μM NaMO4, 1 mM DTT, E64 μM, 2 mM PMSF. The extracts are centrifuged (15,000 *g*, 15 min). Nitrate reductase activity was assayed at 28 °C by measuring NO_2_^−^ production as described in Horchani et al. (2011).

### *In vivo* phosphorus NMR

For each experiment, 0.9 to 1.2 g fresh weight of 4 wpi-old wild type or mutant *M. truncatula* nodules (around 1,400 to 1,800 nodules) were harvested, placed in 10-mm sealed NMR tube, and analyzed as described in Berger et al. (2020b). Oxygen partial pressure in the perfusion medium was established by bubbling mixtures of O_2_ and N_2_ (either 21:79% or 1:99% O_2_:N_2_) into the medium reservoir. ^31^P NMR spectra acquisition conditions and resonance assignments were as in Berger et al. (2020b) in NMR spectrometer (Avance III, Bruker). Data represent the mean of 3 independent biological experiments.

γ-ATP and β-ADP peak areas were estimated after spectral processing with the NMRProcFlow application (Jacob et al. 2017). Given the low signal-to-noise ratio (S/N), the line broadening (LB) parameter was set to 20 to improve this ratio without excessively altering the shape of the signals and a zero-filling (x2) to improve the resolution. A baseline correction using the airPLS method (order 1) (Zhang et al. 2010) was applied to the area of interest ([−4.5 ppm, −8 ppm], Figure S1). Two approaches were then combined to estimate the area of the 2 peaks of interest, using the Rnmr1D package (Jacob 2018). The first performs peak fitting using a mixture of Lorentzian and Gaussian (pseudo-Voigt) as a model. The modeling is imperfect, especially for the β-ADP, because it is highly noisy with an S/N lower than 3 or even 2, but it allows to have a good estimate of the full width at half-maximum (sigma) of the peaks. The second bucket approach consists of integrating the areas within intervals. Given that the area of a bucket only considers a portion of the peak and, moreover, an overlap between peaks generates a mutual contribution, a correction was performed based on the estimate of the full widths at half maximum, as shown in Figure S1.

### Statistical analysis

Pairwise comparisons between 2 groups were performed using the non-parametric Mann-Whitney test. For comparisons involving 3 or more groups, the Kruskal–Wallis test followed by Dunn's post hoc test was applied, using PAST 3 software (https://folk.uio.no/ohammer/past/).

## Results

### Phylogenetic analysis reveals complex evolutionary history of nitrate reductase in Rosids

**Figure 1:**
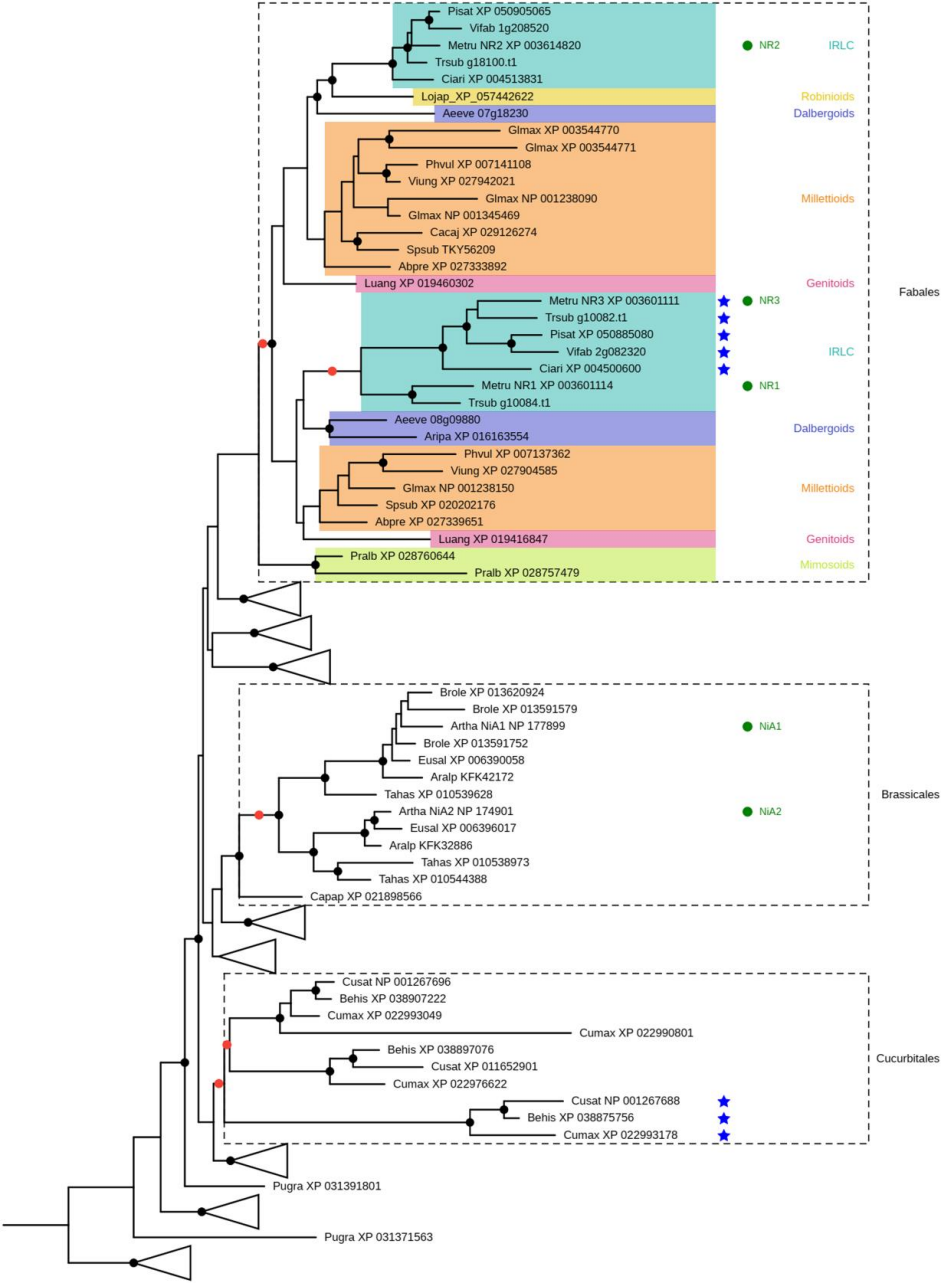
Simplified maximum likelihood phylogenetic tree of NR protein sequences across the Rosids clade. The tree was constructed from 121 NR protein sequences, with the Poales clade used as an outgroup for rooting. Well-characterized sequences, including NIA1 and NIA2 from *A. thaliana* and NR1, NR2, and NR3 from *M. truncatula,* are indicated by a green dot. Blue stars denote NR sequences with a predicted loss of the conserved phosphorylation site. Red dots on selected nodes indicate the duplication events at the origin of the 2 or 3 groups of NR protein sequences found in the Brassicales, Fabales, and Cucurbitales.

Maximum likelihood phylogenetic analysis was conducted to elucidate the evolutionary relationships among NR protein sequences from 10 different orders within Rosids clade (Fig. 1 and Figure S2). NR sequences from the Poales clade served as an outgroup for rooting the phylogenetic tree. The analysis included 121 NR protein sequences from 64 species (Table S2), featuring well-characterized examples such as NIA1 and NIA2 from *A. thaliana* and NR1, NR2, and NR3 from *M. truncatula* (Fig. 1 and Figure S2). In the Rosids clade, at least 1 species with 2 or more NR protein sequences were identified in each order, except for the Sapindales. All orders, except Myrtales, formed monophyletic groups, indicating that the duplication events leading to the emergence of distinct NR genes occurred after the divergence of the various orders within the Rosids clade as marked by the red dot on Fig. 1 and Figure S2. In the Fabales and Brassicales, where NR protein sequences are well characterized for *M. truncatula* and *A. thaliana*, respectively, an ancestral duplication event is evident at the origin of NR1 and NR2 in *M. truncatula* and NIA1 and NIA2 in *A. thaliana* (Fig. 1). Within the Fabales order, this ancestral duplication event occurred after the separation from the *Mimosoids* clade (Fig. 1), making this clade phylogenetically more distant from the other clades included in the analysis. Across Fabales clades, NR1 and NR2 orthologs were identified, with the notable exception of the Robinoid *Lotus japonicus*, which harbors a single NR sequence closely related to MtNR2 (Fig. 1).

Additionally, a more recent duplication event of the *NR1* gene occurred in the ancestor of the inverted repeat-lacking clade (IRLC) within the Fabales (Fig. 1). This duplication event led to the emergence of the *NR3* gene, which is present in all species of the IRLC clade studied so far. Interestingly, the presence of both NR1 and NR3 sequences was observed only in *M. truncatula* and its relative *Trifolium subterraneum* (Fig. 1). In contrast, only the NR3 sequence was identified in other species within the IRLC clade, including *Pisum sativum*, *Vicia faba* and *Cicer arietinum*, suggesting a potential loss of the *NR1* gene in these species. Consequently, a search for orthologs of the 3 *M. truncatula* genes in the genomes of *P. sativum*, *V. faba* and *C. arietinum* yielded results only for *NR2* and *NR3*. A synteny analysis suggests a loss of the *NR1* gene in the genomes of *P. sativum* and *V. faba*, likely occuring in their common ancestor (Figure S3a). The absence of the *NR1* gene in *C. arietinum* can be attributed to the low quality of the genome in that region (Figure S4).

Two ancestral duplication events occurred in the *Cucurbitales,* led to the emergence of 3 *NR* sequences, implying that orders beyond the Fabales may possess additional NR types. This phenomenon is not limited to species capable of nitrogen-fixing symbiosis. Recent duplications have also been observed across various orders, particularly in large-genomes species like *Glycine max,* resulting in an increased number of closely related NR paralogs (Fig. 1).

### Structural and regulatory divergence of NR3-type NR in the IRLC clade

**Figure 2:**
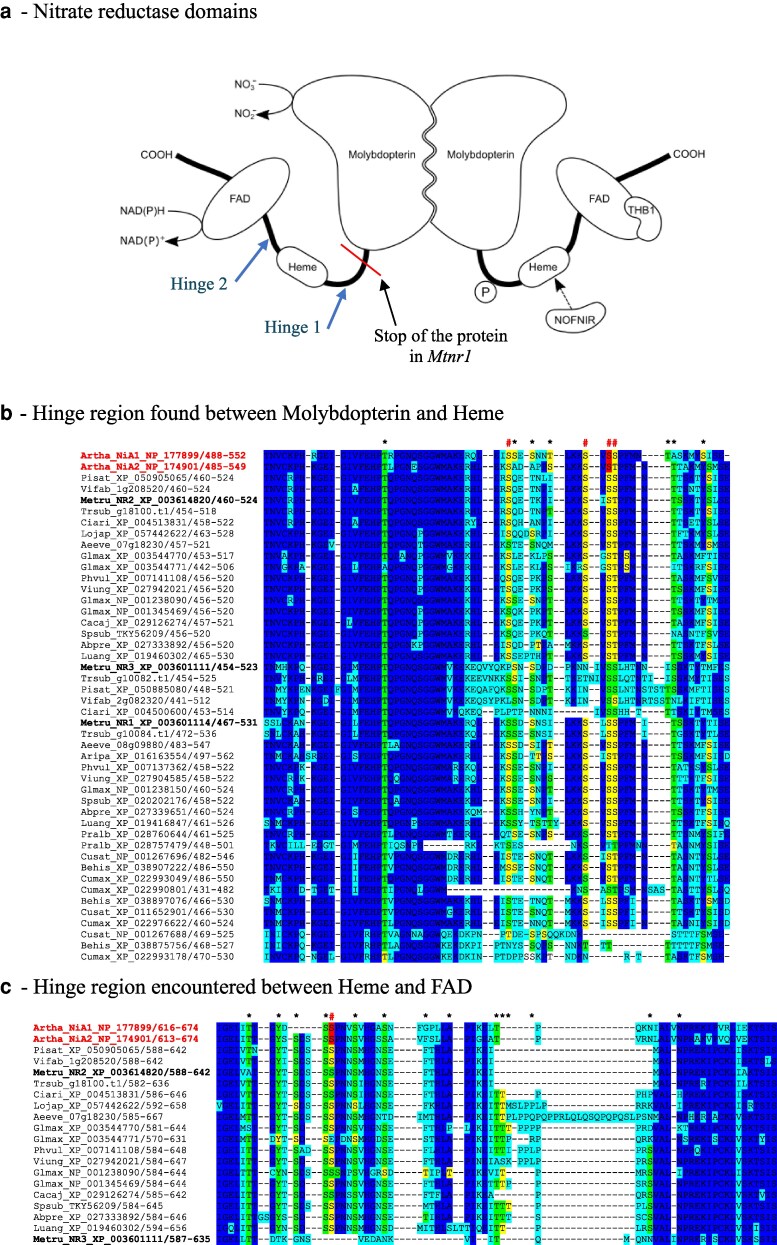
The 2 NR hinge regions and their potential phosphorylation sites. (a) Schematic representation of plant Nitrate Reductase (NR) structure. Each subunit of this protein complex contains 3 prosthetic groups: the cofactors flavin adenine dinucleotide (FAD), the molybdenum cofactor (Moco), and the heme. NR has 2 “hinge” regions situated between the Moco and heme domains (Hinge 1) and between heme and FAD domains (Hinge 2). Phosphorylation site in hinge 1 is represented. (b) Alignment of the NRs protein sequences of *A. thaliana*, *Cucumis sativus* and NRs belonging to the Fabales family in region between the Moco and heme domains, (c) and between the heme and FAD domains. Sequences are labeled with codes explained in Table S2. *A. thaliana* sequences are shown in red, while the names of *Fabales* sequences are presented in black. *M. truncatula* sequences are denoted in bold. Symbols: # indicates highly conserved sites where phosphorylation is predicted in most of the sequences analyzed (>50%). * indicates sites conserved for serine (S), threonine (T), and tyrosine (Y).

Nitrate reductases are molybdoenzymes that function with 3 prosthetic groups: FAD, molybdenum cofactor (Moco), and heme, anchored to distinct domains connected by 2 flexible hinge regions (Campbell 2001) (Fig. 2a). These hinge regions play a key role mediate in post-translational regulation, particularly in response to environmental cues such as light intensity, CO_2_ or O_2_ availability (Kanamaru et al. 1999; Kaiser and Huber 2001). In *A. thaliana*, 2 specific serine residues, Serine 534 in Hinge 1 (between the Moco and heme domains), and Serine 627 in Hinge 2 (between the heme and FAD domains), act as major phosphorylation sites involved in this regulation (Lea et al. 2004; Wang et al. 2010).

In *M. truncatula*, the 3 NR isoforms (MtNR1, MtNR2, and MtNR3) share over 70% sequence similarity (Berger et al. 2020b). However, multiple protein sequence alignments across representatives of *Fabales* and *Cucurbitales*, including *A. thaliana* NR sequences, revealed notable divergence in the hinge regions of NR3-type proteins (Fig. 2b and c). Specifically, Ser534 and Ser627 were conserved across most Fabales NR sequences, but absent in 5 NR3-type proteins: MtNR3, *Cicer arietinum* XP_004500600, *Pisum sativum* XP_050885080, *Vicia faba* 2g082320, and *Trifolium subterraneum* g10082.t1. This non-conservation was further supported by phosphorylation site prediction using Musite (https://www.musite.net/), which confirmed the absence of predicted phosphorylation sites at these positions (Fig. 2b and c).

The lack of conserved post-translational regulatory sites in proteins phylogenetically related to MtNR3 suggests that these NR3-type enzymes may function independently of phosphorylation-based regulation, potentially reflecting functional specialization within the IRLC clade. Intriguingly, 3 NR sequences from *Cucurbitales* (Cusat NP001267688, Behis XP038875756, and Cumax XP022993178) also lacked predicted phosphorylation at analogous sites, suggesting independent loss of this regulatory feature in distinct Rosid lineages, indicative independent evolutionary events in 2 distantly related orders (Fig. 2b and c).

Given that *MtNR3* exhibits a symbiosis-specific expression pattern in *M. truncatula*, notably at the meristem of developing nodules (Berger et al. 2020b), we explored whether *NR3-*type genes from other IRLC species show similar expression profiles. Transcriptomic analyses revealed divergent expression patterns among *NR3*-type genes (Figure S5). In *Cicer arietinum*, *CaNR3* showed stronger expression than *CaNR2* across most tissues analysed, although *CaNR2* was restricted to roots and nodules (Figure S5a; Alves-Carvalho 2015). In *P. sativum*, *PsNR2* was mainly expressed in leaves and roots, whereas *PsNR3* expression peaked in pods, seeds, and nodules (Figure S5b; Kudapa et al. 2018). Expression data for *V. faba* were not available. These findings indicate that, unlike *MtNR3*, *PsNR3* and *CaNR3* are not limited to nodulation-specific expression, arguing against a conserved nodule-specific regulatory role for all NR3-type isoforms.

### Functional analysis of *nr* mutants reveals essential roles of MtNR1 and MtNR2 in nitrate assimilation

**Figure 3:**
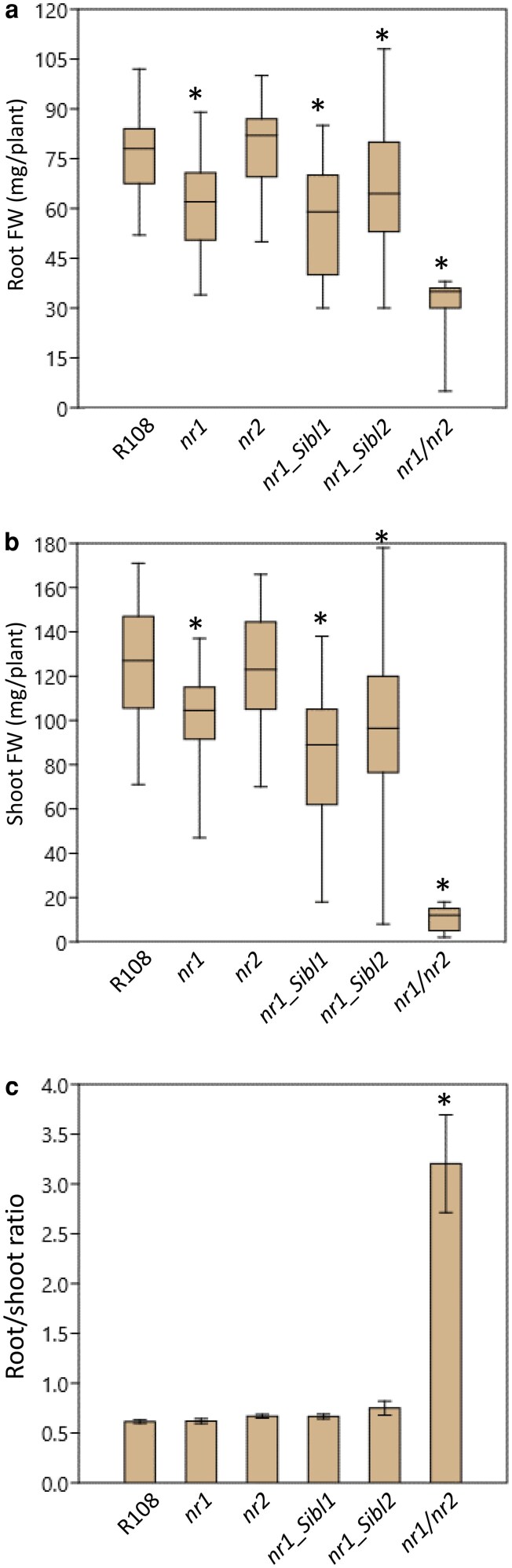
Biomass measurements of different NR mutants growing on nitrate: (a) measurement of root biomass after 2 wks of growth on NO3^−^. (b) Measurement of shoot biomass after 2 wks of growth on NO3^−^. (c) Root/shoot ratio measurement. The asterisks correspond to significant differences determined by the Mann–Whitney test (*P* < 0.05) from 2 independent experiments composed of, at least, 25 plants for each condition.

The *M. truncatula* genome encodes 3 NR isoforms, suggesting possible functional specialization in nitrogen metabolism, NO production and/or hypoxia adaptation. We selected *Tnt1* retrotransposon-tagged mutant lines, each disrupted in a different *NR* gene (Figure S6). The 3 *NR* genes comprise 4 exons (Figure S6). In *nr2* and *nr3*, *Tnt1* are located in the first exon, likely resulting in complete loss of function. In *nr1*, the insertion is located in the fourth exon, potentially allowing the translation of a truncated 480-amino-acid protein (compared to 902 amino acids in the wild-type protein) retaining the molybdopterin domain but lacking the essential heme and FAD domains required for enzymatic activity, making it unlikely to be functional (Figure S6, Fig. 2a). We quantified total NR activity in the shoots and the roots of *nr1*, *nr2* and the segregating double mutant *nr1/nr2* lines (Table 3). The *nr3* line was excluded from this analysis, as *MtNR3* is specifically expressed during symbiotic interaction (Berger et al. 2020b). Two independent sibling lines (*nr1_Sibling1* and *nr1_Sibling2*; Table 2) were included as controls and exhibited the same phenotype as *nr1*, indicating that they harbor the same mutation in the *nr1* gene. NR activity assays revealed a strong reduction in shoot activity in *nr1* (−90%) and *nr2* (−70%) (Table 3). Strikingly, NR activity was nearly abolished in the *nr1/nr2* double mutant, with residual activity representing only 3% and 2% of wild-type levels in shoots and roots, respectively. Shoot and root biomass were approximately 20% lower in the *nr1* mutant (and in *nr1_Sibling1*), whereas the *nr2* mutant showed growth comparable to the wild type (Fig. 3a, b). In the double mutant, biomass was dramatically reduced, with shoots and roots declining by 92% and 60%, respectively. Consequently, the root-to-shoot ratio increased significantly only in the double mutant, reflecting compensatory adjustments typical of severe nitrogen deficiency ((Lopez et al. 2023), Fig. 3c). Phenotypically, double mutant plants exhibited early and rapid chlorosis of the youngest leaves, appearing soon after the emergence of new trifoliate leaves. The chlorosis subsequently spread throughout the canopy, leading to systemic yellowing, growth arrest, and ultimately plant death (Figure S7).

**Table 3:** Measurement of nitrate reductase activity in shoots, roots and nodules of M. truncatula NR mutants. Nitrate reductase activity in shoots and roots was measured in non-inoculated plants grown hydroponically with 1 mM KNO3 as the sole nitrogen source. The nitrate reductase activity is expressed in nmol per g of fresh weight per h. The *nr1_Sibl1^o^* line, which is a knockout for the *NR1* gene with no *Tnt1* insertion in the *NR2* gene was also used as control. Both lines were derived from the same pods as the double mutant. Results are the mean ± SE (*P < 0.05, one-way analysis of variance) of 2 independent experiments composed of, at least, 25 plants for each condition. Each measurement was done in triplicate. NM means Not Measured.

		WT	*nr1*	*nr2*	*nr3*	*nr1_Sibl1*	*nr1/nr2*
NR activity (nmoles/g of FW/h)	Leaves	1,084 ± 258	123 ± 30	306 ± 76	NM	112 ± 14	31 ± 12
	Roots	298 ± 95	27 ± 10	110 ± 20	NM	57 ± 19	5.2 ± 3
	Nodules	229 ± 62	39 ± 21	116 ± 44	224 ± 56	NM	NM

**Figure 4:**
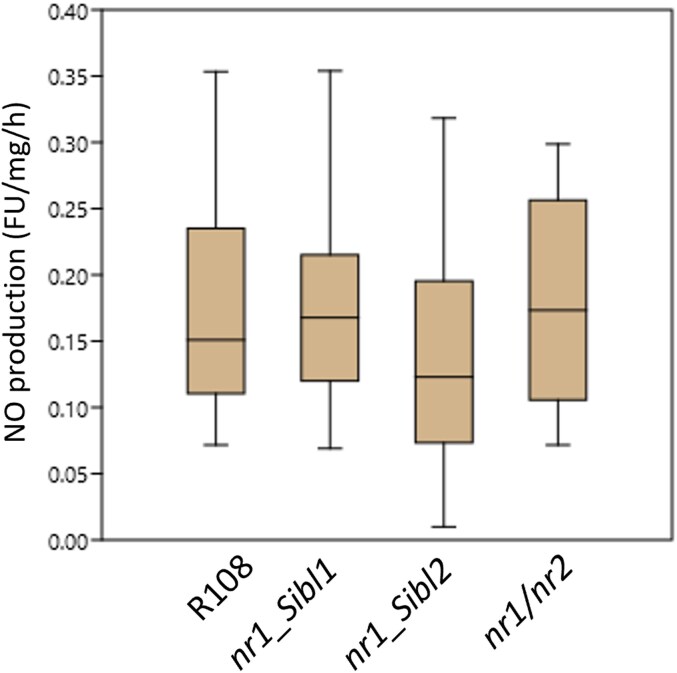
Measurement of NO production from non-inoculated root. The fluorescence intensity of the NO production was measured using the 4,5-diaminofluorescein probe (DAF-2, Sigma-Aldrich). The line within the boxes refers to the median. The asterisks correspond to significant differences determined by the Mann–Whitney test (*P* < 0.05) from 2 independent experiments composed of, at least, 12 plants for each condition. Each measurement was done in triplicate.

To assess whether NR activity contributes to NO production under nitrate-replete conditions, NO production levels were quantified in the roots of wild-type and mutant plants grown with 2 mM NO_3_^−^. NO production in the *nr1/nr2* double mutant was comparable of the wild type (Fig. 4), indicating that, under these conditions, root NO biosynthesis occurs largely independently of NR-derived NO_2_^−^.

Attempts to rescue the double mutant by complementation were unsuccessful. Five independent attempts of *Agrobacterium rhizogenes*-mediated transformations were made using a construct containing the *MtNR2* as described in the Materials and Methods section. Each experiment included approximately 80 seedlings, yielding 12 to 20 confirmed segregating double mutants plants per trial. However, no transgenic double mutant roots capable of growing on nitrate were recovered, indicating a probable failure to restore NR function.

### Single NR mutants show functional redundancy while double mutants display severe nodulation defects

**Figure 5:**
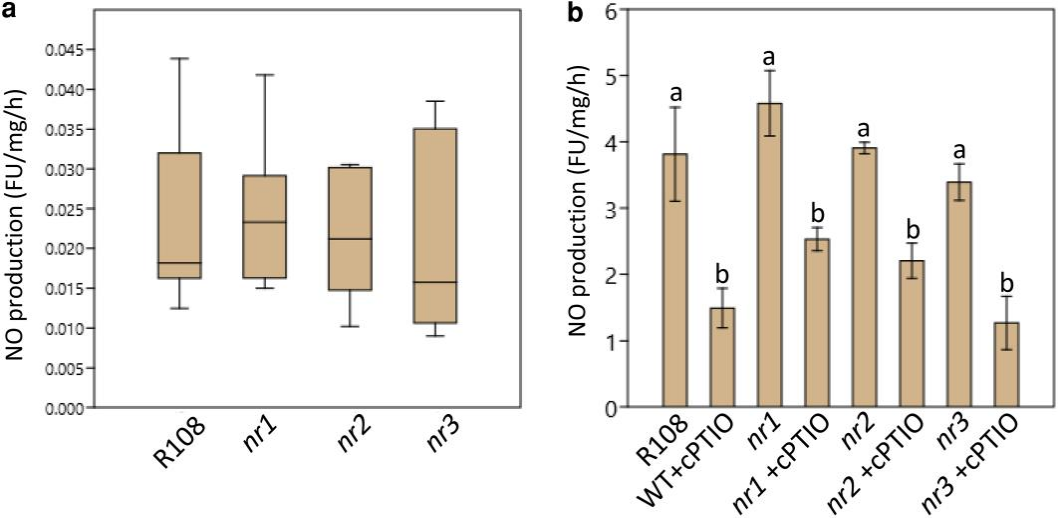
Measurement of NO production from mature nodule. NO production was was quantified by measuring fluorescence intensity using 2 probes: (a) 4,5-diaminofluorescein (DAF-2, Sigma-Aldrich) and (b) FL2A (Strem Chemicals). NO scavenger cPTIO was used with FL2A probe. The box plots display the median (line within the box) and variability among samples. Different letter indicate statistically significant differences (*P* < 0.05, Mann–Whitney test) based on 2 independent experiments, each with at least 12 plants per condition. All measurements were performed in triplicate.

**Figure 6:**
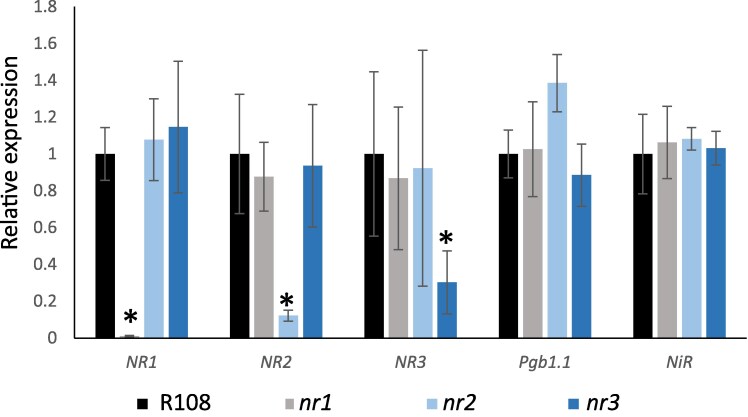
Expression analysis of the 3 *NRs, Pgb1.1 and Nir* genes in nodules from NR mutants. Relative transcript levels were measured by real-time quantitative PCR on 4 wk-old *M. truncatula* nodules, from 4 different genotypes (R108, *nr1*, *nr2* and *nr3*). Results are the mean ± SE. The asterisks (*) correspond to significant differences determined by the Mann–Whitney test (*P* < 0.05) from 2 independent experiments composed of, at least, 10 plants for each condition.

Despite the fact that NR is mainly involved in nitrate assimilation, additional roles have been evidenced. NR activity is required for both the successful establishment and optimal functioning of N_2_-fixing symbiosis in legumes, and it further supports energy homeostasis under low-oxygen conditions via the PNR pathway (Horchani et al. 2011; Berger et al. 2020b). In *M. truncatula*, 3 peaks of *MtNRs* expression and NR activity have been observed: at 10 hours post-inoculation (hpi), during early nodule development (4 d post-inoculation (dpi)), and during nodule maturation (3 to 4 wks post-inoculation (wpi)) (Berger et al. 2020b). To dissect the isoform-specific contributions of NR to the symbiotic process, single *nr1*, *nr2*, and *nr3* mutants were grown in association with *S. meliloti*. The 3 lines exhibited wild-type-like phenotypes with respect to nodule number, shoot biomass accumulation, and nitrogenase activity (acetylene reduction assay) (Figure S8). Biochemical analysis of 4-wk-old nodules revealed a non-significant reduction in NR activity in *nr3* (−3%), an average decrease in *nr2* (−50%), and a pronounced reduction in *nr1* (−87%) relative to the wild type (Table 3). Since NR is a known source of NO during symbiotic interaction (Berger et al. 2020b), we assessed NO production in 4-wk-old nodules. Fluorescent-based quantification of NO production was not altered in any of the 3 *nr* mutants (Fig. 5), regardless of whether the DAF2 probe (Fig. 5a) or the FL2A probe (Fig. 5b) was used. As expected the NO scavenger cPTIO reduced fluorescence in FL2A-stained nodules (Fig. 5b), confirming the specificity of the measurement. We next analyzed the expression of *MtPhytoglobin1.1* (*MtPgb1.1*), a key regulator of nodule NO homeostasis and a reliable transcriptional marker of NO levels, as its expression is directly induced by NO (Berger et al. 2018, 2020a). *MtPgb1.1* transcript levels were unchanged in all *nr* mutants (Fig. 6), further indicating that nodule NO homeostasis remains unaffected in each *nr* mutant. Quantitative PCR analysis revealed that the loss of a single *NR* gene did not induce compensatory upregulation of the remaining NR isoforms (Fig. 6). In addition, transcript levels of *MtNiR*, which are known to increase in *nr* mutants of *Nicotiana plumbaginifolia* (Faure et al. 1991) remained unchanged. These results support the notion of functional redundancy among *M. truncatula* NR isoforms in nodules and suggest that individual gene loss does not trigger transcriptional reprogramming of the nitrate assimilation pathway.

**Figure 7:**
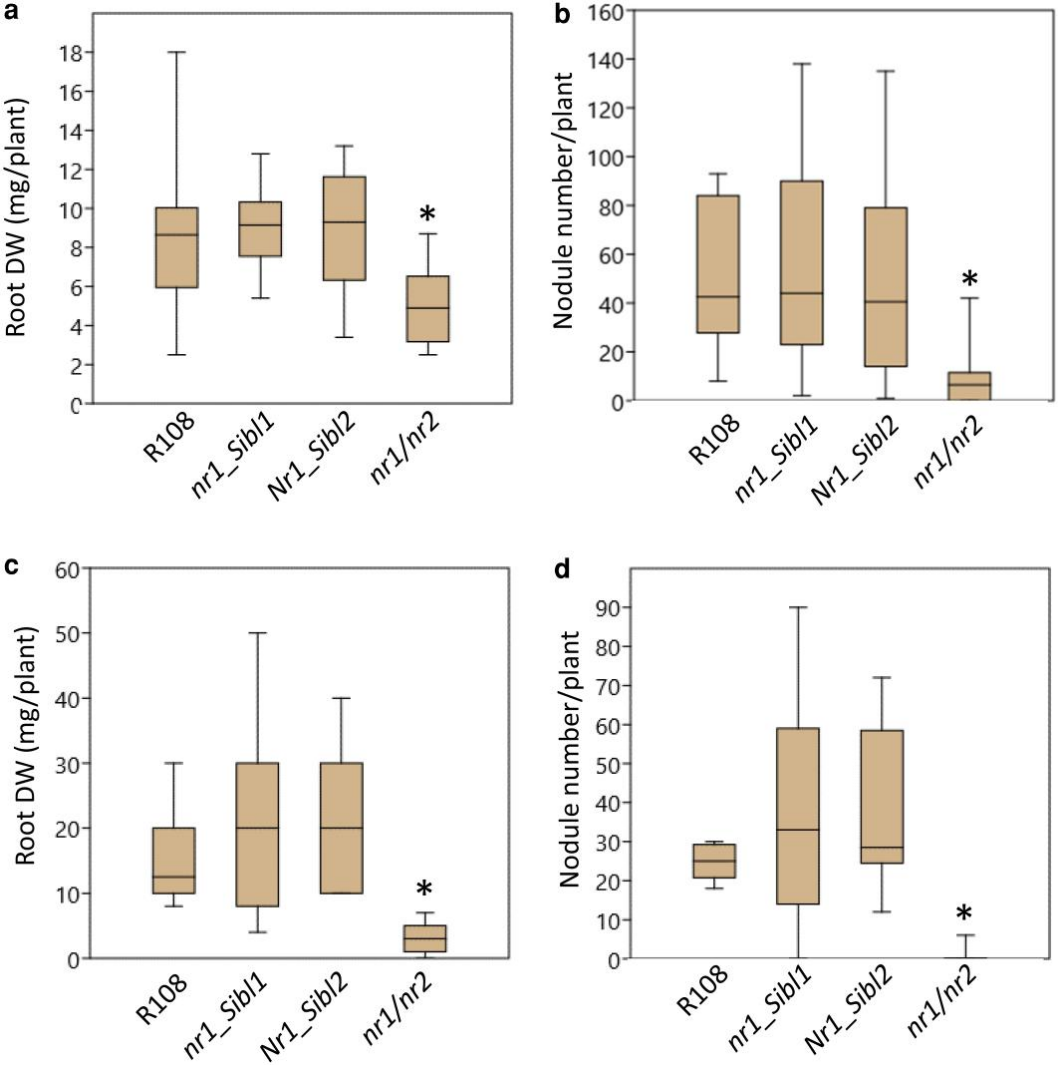
Phenotype of *M. Medicago* NR mutants during the nodulation process. Boxplot representation of root dry weight and nodule number measured on hydroponic culture of segregating double mutant after 7 d (a and b) and 4 wks (c and d) post inoculation with *S. meliloti*. The line within the boxes refers to the median. The asterisks correspond to significant differences determined by the Mann–Whitney test (*P* < 0.05) from 2 independent experiments composed of, at least, 15 plants for each condition. Note that at 7 dpi, nodule counts were performed on scanned root images, where early primordia can be difficult to distinguish from emerging lateral roots, potentially leading to an overestimation of nodule numbers at this stage. At 4 wpi (panel d), counts were performed on fully developed nodules only, allowing unambiguous identification and quantification of mature nitrogen-fixing structures.

To further investigate, we analyzed a *nr1/nr2* double mutant alongside segregating sibling lines (*nr1_Sibl1* and *nr1_Sibl2*) inoculated with *S. meliloti*. The double mutant displayed a severed nodulation phenotype: an 85% reduction of nodule number at 7 dpi, and a 98% reduction at 4 wpi (Fig. 7b and d). At 7 dpi, root dry weight was reduced by 50% and shoot DW by only 25% (Fig. 7a; Figure S9a). At 4 wpi, both root and shoot biomass were reduced by ∼70% relative to controls (Fig. 7b; Figure S9b). Due to the near absence of nodules, N2-fixation capacity and NO production could not be assessed in the double mutant.

### NR activity modulates nodule resilience to hypoxic stress and post-stress recovery

To explore the role of NR in the metabolic response of *M. truncatula* nodules to hypoxia, we evaluated the impact of either short-term (3 h) or medium-term (5 d) low-oxygen treatments on energy status and metabolic profiles in wild-type and single *nr* mutant plants. Given the known involvement of NR in the PNR respiration pathway, we investigate the effect of disruption of individual *NR* genes on energy metabolism and recovery under hypoxic stress on nodules.

**Figure 8:**
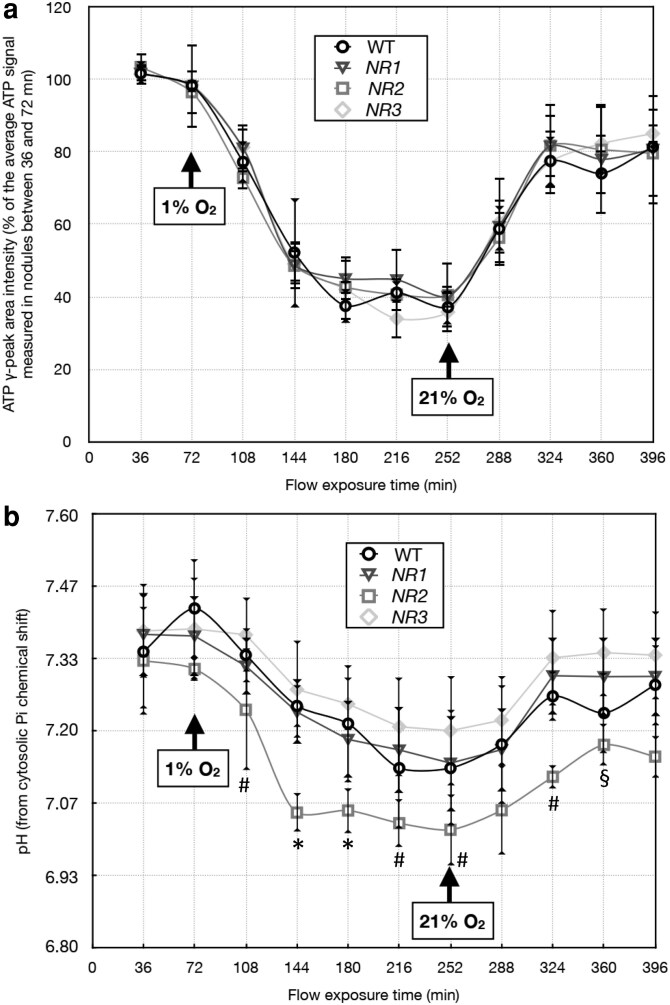
Evolution of ATP content and cytosolic pH in *M. truncatula* nodules under normoxic and hypoxic conditions. (a) Relative intensity of the ATP γ-peak area (measured by 31P-NMR) over time in nodules from 4-wk post-inoculation (wpi) *M. truncatula* plants, either wild-type (WT) or mutants (*nr1*, *nr2*, *nr3*). Values are expressed as a percentage of the total ATP signal measured in WT at 36 minutes (set as 100%). (b) Cytosolic pH evolution, calculated from the chemical shift of cytosolic inorganic phosphate (Pi) using 31P-NMR. For each experiment, 0.9 to 1.2 g of fresh nodule tissue (approximately 1,400 to 1,800 nodules) was placed in a 10-mm closed NMR tube and perfused with medium either saturated with air (normoxia, 21% O2) or under hypoxia (1% O2). Data represent means (± SD) of 3 independent in vivo experiments. Statistical analysis was conducted using a one-way ANOVA (genotype × time), followed by Dunn's post hoc test (*P* < 0.05). Asterisks (*) indicate significant differences between *nr2* and both WT and *nr3* genotypes; hashtags (#) indicate differences between *nr2* and *nr3*; and (§) denotes differences between *nr2* and both *nr1* and *nr3* genotypes.

For short-term low oxygen treatment, we used a perifusion system adapted to NMR spectrometer (Berger et al. 2020b). We first followed the effects of in vivo transition from normoxia (21% O_2_) to hypoxia (1% O_2_), and vice versa, on ATP level and cytosolic pH value of 4 wpi nodules. As shown in Fig. 8, under normoxia, ATP levels are similar in WT and *nr*-mutants (Fig. 8a), and cytosolic pH values are around 7.35 ± 0.1 (Figure 8b). Transition from normoxia to hypoxia leads to an important reduction in ATP content (50%) and a significant acidification of cytoplasmic pH (7.00 to 7.20) after 3 h of hypoxia. Interestingly, while ATP levels drop similarly in WT and mutants nodules, the pH value decreases significantly faster and more sharply in *nr2* nodules (7.00) than in the others (7.12 to 7.20). This means that, although the overall ATP content is not different, ATP turnover and energy metabolism are more affected in *nr2* than in other nodules. Upon return to normoxia, ATP levels recovered similarly in all lines over the following 2.5 hours. However, cytosolic pH remains significantly lower in *nr2* (7.20) compared to the other 3 (7.30 to 7.35), indicating a delayed restoration of cytosolic homeostasis in *nr2* nodules. Taken together, these data reveal that energy metabolism of *nr2* nodules is more sensitive to short-term hypoxia and recovery than that of WT, *nr1* and *nr3* nodules.

**Figure 9:**
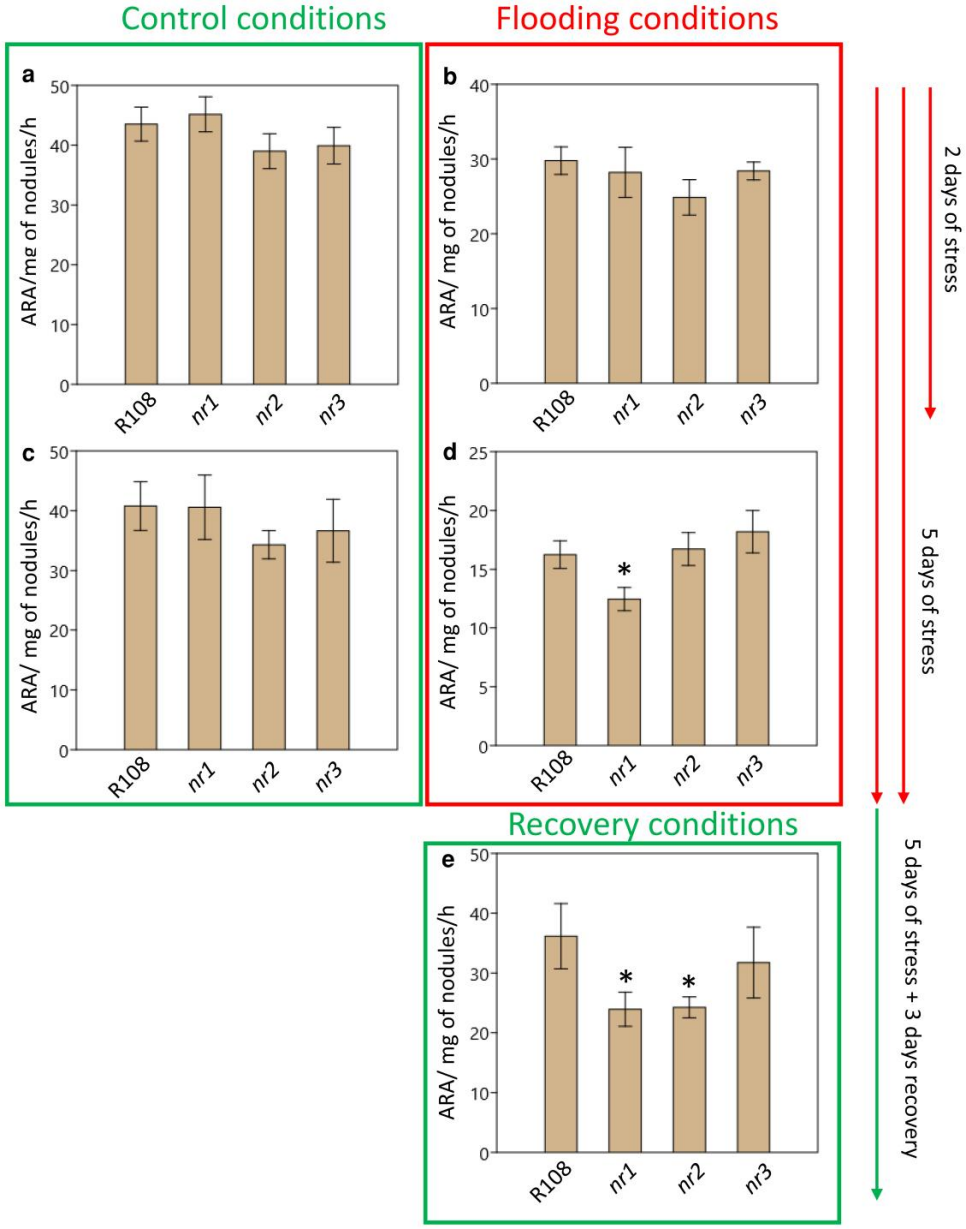
Nitrogen-fixing capacity of the different *M. truncatula nr* mutants: (a) Nodule number was determined on plants growing in plate with Fahraeus medium at 14 d post inoculation. (b) Plant biomass was measured on the shoot of the plant used for acetylene reduction assay (ARA). (c) The nitrogen-fixing capacity was measured by the ARA expressed in nmol of ethylene (C2H4) reduced per mg of nodules and per hour, from plant nodules at 4 wks post inoculation (wpi). The asterisks correspond to significant differences determined by the Mann–Whitney test (*P* < 0.05) from 2 independent experiments composed of, at least, 9 plants for each condition.

To assess longer-term effects, root systems of inoculated plants were subjected to flooding for 2 or 5 d, followed by a 3-d recovery period. Nitrogen fixation capacity (ARA measurement) declined significantly by approximately 30% after 2 d of flooding (Fig. 9a and 9b) and by 60% after 5 d of flooding in WT plants (Fig. 9c and 9d), with partial recovery (82% of the control level) observed after the recovery period (Fig. 9e). The 3 single *nr* mutants showed no notable phenotypic differences after 2 d of flooding. However, after 5 d, the *nr1* mutant exhibited a significantly greater reduction in nitrogen fixation activity than WT and the other genotypes (Fig. 9d). Furthermore, during the recovery phase, both *nr1* and *nr2* mutants displayed impaired reactivation of nitrogenase activity, with ARA levels remaining 21% and 24% below WT values, respectively (Fig. 9e). These results suggest that reduced NR activity resulting from *nr1* or *nr2* mutation compromises the resilience of nodules to prolonged hypoxic stress and impairs post-stress recovery.

## Discussion

### Phylogenetic and structural evidence for the specialization of NR3 in the Fabales clade

In *M. truncatula*, *MtNR1* and *MtNR3* are only 20 kb apart (Berger et al. 2020b), potentially the duplication might be generated by unequal crossing-over. While 3 *NR* genes are present in *M. truncatula* and *Trifolium subterraneum*, only 2 were found in *P. sativum*, *V. faba* and *C. arietinum* indicating that *NR* gene copy number and organization may vary among legume species. Notably, the *NR1* ortholog is absent in these plants. Given that IRLC legumes form indeterminate nodules expressing Nodule Cysteine-Rich peptides (NCR) involved in terminal bacteroid differentiation (Czernic et al. 2015), positive selection observed in the NR3 lineage suggest functional divergence. Sequence alignments revealed modifications in conserved hinge regions of NR3-type proteins in 5 Fabales species. Phosphorylation of Ser534 in *A. thaliana* Hinge 1 promotes NR-14–3-3 protein complex formation, increasing proteolytic susceptibility (Fig. 2b) (Lea et al. 2004; Lillo et al. 2004), while Ser627 phosphorylation in Hinge 2 provides rapid activation for excessive nitrate reduction (Wang et al. 2010). The divergence in NR3 suggests an alternative regulatory mechanism, potentially conferring resistance to degradation as reported by Lillo et al. (2004) and enabling specific roles during nodule senescence. *MtNR3* expression is induced exclusively during symbiosis and repressed by nitrate, contrasting with *NR1* and *NR2* induced by nitrate (Berger et al. 2020b). However, the *Mtnr3* mutant displayed no phenotypic defects during symbiosis or senescence (Figure S8), possibly due to low and spatially restricted expression, consistent with unaltered NR activity in roots and nodules (Table 3).

However, in *P. sativum* and *C. arietinum*, NR3-type genes are expressed in multiple tissues, suggesting no strict association with nodulation such as for *MtNR3*. A third *NR* gene also exists in Cucurbitales, with similar loss of phosphorylation sites (eg, *C. sativus* Cusat_NP_001267688). This structural similarity, despite the absence of symbiotic ability in Cucurbitales, suggests a broader evolutionary advantage of constitutively active NRs. Supporting this, mutation of conserved serines in NRs from Nicotiana and Oryza species rendered enzymes constitutively active, affecting nitrogen assimilation and plant development (Lillo et al. 2003; Han et al. 2022). The emergence of NR3-type proteins lacking key regulatory phosphorylation sites may therefore represent an adaptive strategy favouring sustained NR activity under specific ecological or physiological conditions. It remains an open and intriguing question whether species that have retained NR3 at the expense of NR1 during evolution, such as *P. sativm* and *C. arietinum*, have undergone distinct functional adaptations associated with this shift.

### Loss of MtNR1 and MtNR2 severely compromises nitrate assimilation and plant survival under exclusive nitrate supply

NR activity is most strongly reduced in *nr1*, which retains only about 10% of wild-type levels, similar to the Arabidopsis *nia2* mutant (Wilkinson and Crawford 1991), but, unlike *nia2*, *nr1* mutant plants show a clear reduction in shoot and root biomass under nitrate nutrition. *nr2* mutants, with 30% residual activity, display normal growth. It is noteworthy that the combined effect of *nr1* and *nr2* mutations on NR activity exceeded 100% in leaves, roots, and nodules. This superadditive effect suggests that both NR1 and NR2 must be present simultaneously to achieve full enzymatic activity, indicating a potential cooperative or synergistic interaction between the 2 isoforms in maintaining total NR activity. However, to our knowledge, no similar observations have been reported in the literature that would allow further discussion of this phenomenon. In the *nr1/nr2* double mutant, NR activity dropped to 3% in shoots and 2% in roots, levels equivalent to the 0.5% activity reported for the Arabidopsis *nia1/nia2* mutant (G′4-3 mutant) (Wilkinson and Crawford 1993) and to the complete loss of NR activity in the NR-null mutant (Wang et al. 2004). Unlike the Arabidopsis G′4-3 double mutant, which shows very poor growth on media with nitrate, the Medicago double mutant *nr1/nr2*, as a Arabidopsis NR-null mutant, does not grow under the same conditions. This suggests that the remaining 2 to 3% NR activity detected is likely due to background noise from the measurement method. Furthermore, contrary to Arabidopsis NR-null mutant and in spite of numerous attempts of growth experiments with different sources of nitrogen, it was not possible to get to the point of flowering and seed production in the double mutant seedlings that are knockout on the *MtNR1* and *MtNR2* genes. The seedlings show poor survival and produce only a few leaves, which rapidly undergo necrosis (Figure S7).

Genetic complementation of the *nr1/nr2* double mutant was attempted through transient *A. rhizogenes*-mediated transformation to reintroduce the *MtNR2* gene at the root level. Despite numerous attempts, no transgenic roots capable of sustained growth on nitrate were recovered, indicating that NR function was not restored. Rather than undermining the strength of our conclusions, this outcome is fully consistent with previous reports highlighting the challenges of complementing NR-deficient mutants, which are often attributed to post-transcriptional gene silencing or co-suppression triggered by transgene overexpression (Napoli et al. 1990; van der Krol et al. 1990; Vaucheret et al. 1990; Wilkinson and Crawford 1991). The robustness of our conclusions rests on the severity and reproducibility of the phenotypes across independent mutant lines, on quantitative NR activity measurements across multiple organs, and on the biologically coherent gradient of phenotypic severity observed from single to double mutant combinations, a pattern fully consistent with the known consequences of NR deficiency across plant species. We acknowledge that the characterization relies on single alleles for each gene, and that future work using additional independent alleles or alternative complementation strategies would further consolidate these findings. Nevertheless, the biochemical, physiological, and symbiotic phenotypes collectively provide a robust and internally consistent framework that firmly establishes the essential roles of MtNR1 and MtNR2 in nitrate assimilation and plant growth under nitrate-supplied conditions.

### Nitrate reductases are essential for nodulation in M. truncatula

NR activity is typically present in legume root nodules of *L. japonicus* (Kato et al. 2003), *G. max* (Kanayama et al. 1999) and *M. truncatula* (Horchani et al. 2011; Berger et al. 2020b). The localization of NR mRNA within the infected regions of pea root nodules (Kato et al. 2003) and *M. truncatula* nodule (Horchani et al. 2011) suggest a specific function in this organ. Previous studies have provided evidence for a role of NR activity in N_2_-fixing symbiosis and nodule energy metabolism (Horchani et al. 2011; Aridhi et al. 2020; Chammakhi et al. 2022). However, our work did not reveal any detectable phenotype affecting the establishment or functioning of the N_2_-fixing symbiosis in the single *nr* mutants (Figure S8). The most striking phenotype emerged in the *nr1/nr2* double mutant, which displayed severe nodulation defects (85% reduction at 7 dpi, 98% at 4 wpi) despite retaining *MtNR3* expression. This dramatic phenotype, absent in single mutants, demonstrates that NR activity is required for successful symbiosis establishment and/or maintenance. It is noteworthy to mention that MtNR3 failed to functionally compensate this defect. Although we could not identify the specific role of NR3, it would be interesting to analyze the function and role of NR3-type enzymes in plants that have naturally lost the *NR1* gene, such as pea or chickpea.

In previous work, Berger *et al*. (Berger et al. 2020b) reported that silencing of *MtNR1*, *MtNR2*, or both genes simultaneously using a nodule-specific RNA interference (RNAi) strategy resulted in approximately a 50% reduction of NR activity, accompanied by a corresponding decrease in NO production (Berger et al. 2020b). Similarly, overexpression of *NIA1*, and to a lesser extent *NIA2*, in *Arabidopsis* was shown to promote NO accumulation, suggesting a gene-specific involvement of NR in NO synthesis (Costa-Broseta et al. 2021). However, no significant alteration in NO production was detected in the nodules of the single mutants. This discrepancy suggests the existence of compensatory pathways or alternative NO sources could buffer NO levels in the absence of individual NR isoforms. Indeed, the mechanisms of NO production in plants remain complex and are still not fully resolved. While NR is a key contributor, it is not the sole enzymatic source of NO (Berger et al. 2018; Kolbert et al. 2019). In nodules, NO production cannot be exclusively attributed to the plant; bacterial symbionts are known to contribute substantially (Horchani et al. 2011; Salas et al. 2021). Previous work has shown that in *M. truncatula* and soybean nodules, the bacterial partner produces between 33% to 90% of NO (Sánchez et al. 2010; Horchani et al. 2011). We can therefore postulate that in *nr* mutants, nitrate is reduced to nitrite mainly in bacteroids. This would explain why NO production did not vary between the mutants and the wild-type in this study? Additional factors may contribute to the discrepancies with previous RNAi studies. In RNAi studies, NR gene silencing is restricted to the root system due to the use of *A. rhizogenes* transformation technic, whereas in our mutant lines the loss of NR activity affects the entire plant. NR deficiency is known to trigger major metabolic alterations. Classic studies in NR-deficient plants reported leaf chlorosis, strong reduction in CO_2_ fixation, accumulation of starch, and decreased levels of malate, sucrose, and chlorophyll, despite unchanged nitrate pools and similar total reduced nitrogen levels (Saux et al. 1987; Wang et al. 2004). These data highlight that the consequences of NR deficiency extend beyond nitrogen assimilation per se and reflect a broader metabolic imbalance affecting photosynthesis, carbon partitioning, and energy metabolism. Such alterations provide a clear mechanistic basis for the reduced biomass observed in the *nr1/nr2* mutant. In the context of the symbiosis, these metabolic perturbations are likely to be exacerbated. Functional nodules rely on a tight metabolic coupling between plant and bacteroid partners, in which sucrose imported from source leaves fuels bacteroid respiration and N_2_ fixation, while fixed nitrogen is returned to the plant as ammonium or amino acids. Although *nr1/nr2* plants retain some capacity to initiate nodule primordia under hydroponic conditions, these structures could fail to mature into nitrogen-fixing organs. This suggests that while early Nod factor signaling remains partially functional, the progression of nodule development is likely limited by the strong impairment of photosynthesis associated with NR deficiency-induced chlorosis. Additionally, knowing that NRs are essential components of the plant phytoglobin–NO (Pgb-NO) respiration pathway that enables nodule function under microoxic conditions (Horchani et al. 2011), we propose that the complete loss of NR1 and NR2 function compromises nodule bioenergetics beyond the compensatory capacity of alternative systems.

### NR activity contributes to nodule metabolic resilience under hypoxia

Plant NR contributes not only to nitrogen assimilation but also to energy metabolism, cytosolic pH homeostasis, and the coordination of carbon and nitrogen metabolism in nodules under hypoxic conditions (Berger et al. 2018; Berger et al. 2020b, 2021). Under low-oxygen conditions, NR has also been shown to allow functions beyond nitrogen assimilation, including the regeneration of NAD^+^ and the prevention of cytosolic acidification to life-threatening levels (Allègre et al. 2004). As previously reported in nodulated *Medicago sativa* subjected to 5 d of flooding, Pgb-NO respiration is activated throughout the root system and contributes to maintaining cellular energy status under hypoxic stress (Aridhi et al. 2020). More recently, this pathway was shown to be induced in roots or further enhanced in nodules in response to declining oxygen availability (Chammakhi et al. 2025). The lack of phenotype in *nr1* mutants under control conditions contrasts with their pronounced sensitivity to prolonged flooding and the subsequent recovery phase (Fig. 9), underscoring a role for NR1 in stress resilience likely associated with the Pgb-NO respiration pathway. The delayed recovery of *nr1* and *nr2* mutants after hypoxic stress may be due to a reduced capacity to counteract oxidative stress during the reoxygenation phase (Pucciariello and Perata 2021; Liu et al. 2022). Previous studies have shown that NR1, like Phytoglobin1 (Pgb1), is upregulated during hypoxia and is part of the conserved core hypoxia response gene set (Rovere et al. 2023). It is therefore plausible that NR enzymes contribute to hypoxia tolerance, potentially through roles in redox regulation and/or metabolic adaptation. This may partly explain why, in the absence of both NR1 and NR2, the symbiotic interaction fails to give rise to functional nitrogen-fixing nodules. It was recently shown that prolonged flooding led to increased NO production in roots but a decreased 1 in nodules (Chammakhi et al. 2025). This was accompanied by elevated NR activity and upregulation of genes involved in the Pgb-NO respiration pathway, indicating a role in sustaining energy metabolism under hypoxic conditions. Functional analysis using *M. truncatula nr1* and *nr2* mutants revealed that both genes contribute to ATP regeneration during the early phase of flooding (Chammakhi et al. 2025).

Collectively, these findings underscore the critical role of nitrate reductases (NRs) in the hypoxic response of legume root systems and highlight the greater sensitivity of nodules compared to roots to oxygen deprivation. Nonetheless, the compromised recovery of nitrogen fixation activity after hypoxic stress in *nr1* and *nr2* nodules points to a crucial, possibly non-catalytic, role for NR in stress adaptation, perhaps through participation in redox buffering, NO signaling, or interaction with other components of the Pgb–NO cycle. Accumulating evidence indicates that hypoxic tolerance relies not only on the regulation of NO production but also, critically, on its efficient scavenging (Gibbs et al. 2015; Hsiao et al. 2024). Enhanced NO removal capacity has been associated with the stabilization of key hypoxia-responsive transcriptional regulators by limiting their degradation through oxygen- and NO-dependent proteolytic pathways (Gibbs et al. 2015). These observations reinforce the idea that the balance between NO generation and removal is a central determinant of stress adaptation. Accordingly, NR function should be considered within the broader Pgb–NO regulatory network, where its contribution to NO homeostasis, potentially beyond its enzymatic activity, plays a decisive role in coordinating the plant's ability to withstand and recover from oxygen deprivation.

In line with this framework, the absence of functional nodules in the *nr1/nr2* double mutant indicates that NR activity is indispensable for an effective symbiosis under microoxic conditions, which are inherent to nodule function. In particular, the loss of NR is expected to compromise the Pgb–NO respiration pathway, a key mechanism sustaining energy production under low oxygen by coupling NO turnover to mitochondrial electron transport.

As a consequence, impaired NO cycling would directly limit ATP supply and redox buffering capacity required for nodule initiation and function. Given that nodules operate under tightly controlled microoxic conditions to protect nitrogenase while maintaining respiration, disruption of this alternative respiratory pathway is likely to be especially detrimental. Altogether, these observations support the view that the absence of nodulation in the double mutant is not merely a consequence of defective nitrogen metabolism, but rather reflects a failure to sustain the Pgb–NO-dependent metabolic and signaling network required to establish and maintain symbiosis under microoxic conditions.

These findings collectively reframe our understanding of NR function in legumes, demonstrating that these enzymes serve as molecular bridges connecting nitrogen metabolism, energy homeostasis, and stress adaptation within the unique physiological context of symbiotic nitrogen fixation. Future research should focus on elucidating the specific mechanisms underlying NR contributions to nodule bioenergetics and stress tolerance, knowledge that will be essential for developing climate-resilient legume crops capable of maintaining high nitrogen fixation efficiency under suboptimal conditions.

## Supplementary Material

kiag377_Supplementary_Data

## Data Availability

The authors declare that the research was conducted in the absence of any commercial or financial relationships that could be construed as a potential conflict of interest.
