# Supplementary material for: Functional divergence and symbiotic significance of nitrate reductase isoforms in Medicago truncatula
Source: Plant Physiol. 2026 Jun 16;201(3):kiag377. doi: 10.1093/plphys/kiag377 (PMC13332110; doi:10.1093/plphys/kiag377)
Supplement: kiag377_Supplementary_Data [file kiag377_supplementary_data.zip › Table S1 Bosseno et al. 2026.docx]

**Table S1. List of primers used for *Tnt1* insertion verification, cloning and quantitative RT-PCR analysis.**

| *Primers for Tnt1 insertion verification and cloning of MtNR2 ORF sequence for complementation* | | | | | |
| --- | --- | --- | --- | --- | --- |
| ***Name*** | **Forward primer** | | ***Name*** | | **Reverse primer** |
| ***a80F*** | TACTATTGCAACGAGAGGA | | ***a80R*** | | GGACAATGATCCATGCTG |
| ***a317F*** | GGTTTCATCACTCCAGTTCC | | ***a317R*** | | CAATCCTCTTAAGCCATTTCAC |
| ***a309F*** | CACCACGGCTTCATAACTC | | ***A309R*** | | GATTCGCTTCAACCATTTCAC |
| ***LTR4*** | TACCGTATCTCGGTGCTACA | | ***LTR5*** | | GCCAAAGCTTCACCCTCTAAAGCCT |
| ***a135*** | CTCGAGACCATCCTTCCACCGA | | ***a136*** | | CTCGTGATGTTATTCTTGCT |
| *Primers for RT-PCR analysis* | |  | |  | |

| **Name** | **Forward primer** | **Reverse primer** | **ID in Mt4.0** | **Reference** |
| --- | --- | --- | --- | --- |
| ***Mtc27*** | TGAGGGAGCAACCAAATACC | GCGAAAACCAAGCTACCATC | Medtr2g436620 | Del Guidice et al., 2011 |
| ***a38*** | TCGTGGTGGTGGTTATCAAA | TTCAGACCTTCCCATTGACA | Medtr4g109650 | Del Guidice et al., 2011 |
| ***Pgb1.1*** | CGGTAAAGTTACGGTCAGAG | AAGTGCAAACTTTGTCACCT | Medtr4g068860 | Berger et al., 2020 |
| ***NR1*** | GTTCAGTTTGCAGTAAAGCC | ATACATACAGCGTCGTACTC | Medtr3g073180 | Horchani et al., 2011 |
| ***NR2*** | CCACCTATGATTCAATTTGCTG | TCTATTACTTGCCCTAGAACAC | Medtr5g059820 | Horchani et al., 2011 |
| ***NR3*** | TTTAACCACAGACACCAAAGG | CTCACGTGGGTTAATTAAAGCC | Medtr3g073150 | This work |
| ***NiR*** | AAATGGTAAGGCTACTGAAGG | CTACAATAGGCACCAAGTCC |  | This work |
